# Identification of Patients With Elevated Urine Albumin–to-Creatinine Ratio Levels in a Type 2 Diabetes Mellitus Cohort Based on Data Submitted by Patients via a Smartphone App (SMART-Finder): Protocol for an Observational Study

**DOI:** 10.2196/44996

**Published:** 2023-04-05

**Authors:** Christian Mueller, Markus Schürks, Thomas Neußer, Uschi von der Osten, Daniela Weihermüller, Ira von Arnim, Stephan Martin

**Affiliations:** 1 Bayer Vital GmbH Leverkusen Germany; 2 Smartpatient GmbH München Germany; 3 Verbund Katholischer Kliniken Duesseldorf (VKKD) und Westdeutsches Diabetes- und Gesundheitszentrum (WDGZ) Düsseldorf Germany

**Keywords:** app-based documentation, chronic kidney disease, prevalence, MyTherapy, type 2 diabetes mellitus, urine albumin-to-creatinine ratio screening

## Abstract

**Background:**

Despite effective treatment options, chronic kidney disease (CKD) has become a major cause of mortality worldwide due to the ever-increasing number of patients with type 2 diabetes mellitus (T2DM). Guideline-compliant, at least, annual screening of patients with T2DM is crucial to prevent renal disease progression. However, data on the prevalence of CKD in patients with T2DM and on screening frequency are limited. SMART-Finder is the first study to exclusively use data provided directly by patients via an adherence app to collect information on the prevalence of CKD, risk factors, disease management, and quality of life of patients with T2DM in Germany.

**Objective:**

The primary objective of this study is to determine the proportion of patients with T2DM and an elevated urine albumin-to-creatinine ratio (UACR; albumin-to-creatinine ratio stage A2 and A3) at baseline and after 12 (±3) months. Secondary objectives include the proportion of patients who remain in or switch to another albumin-to-creatinine ratio classification category after 12 months, information on quality of life, disease awareness, and adherence rates, as well as the proportion of patients without any UACR-screening data. Recruitment occurs via push notification among MyTherapy app users with T2DM.

**Methods:**

This is a single-arm, retrospective/prospective, observational, digital, patient-centered cohort study, with recruitment and data documentation via a health app. Required routine laboratory data are provided by treating physicians to their patients for data entry. The study population includes adult patients with T2DM documenting their data in the MyTherapy app using their own smartphone or tablet. Study participants are provided with a specifically developed electronic case report form containing questions on demographic and general data, quality of life, disease awareness, and laboratory values including estimated glomerular filtration rate, UACR, hemoglobin 1Ac, and blood pressure. Apart from demographic and general data, all data are collected at baseline and 12 months after the last UACR assessment. An automatically generated push notification reminds participants of the second data entry. The extracted and pseudonymized data are analyzed descriptively.

**Results:**

The enrollment period for this study started in February 2023 and shall end after 12 months or after the enrollment of 5000 patients. An interim analysis is planned 3 months after the inclusion of the first patient and the final analysis after 12 months of follow-up.

**Conclusions:**

Overall, the study will contribute to minimizing the existing data gap on the prevalence of CKD in patients with T2DM in Germany, provide important insights into the current disease management of patients with T2DM in everyday clinical practice in Germany, and support guideline-based care for the participating patients.

**International Registered Report Identifier (IRRID):**

PRR1-10.2196/44996

## Introduction

Chronic kidney disease (CKD), a common comorbidity of the ever-increasing number of patients with type 2 diabetes mellitus (T2DM), is a leading cause of death worldwide [1-9]. It is usually characterized by persistently decreased estimated glomerular filtration rate (eGFR) or persistently elevated urine albumin excretion, is often progressive and may finally lead to end-stage renal disease [10-13].

At the same time, effective therapeutic approaches are available, which, if applied early and consistently, can favorably influence or delay the development or progression of kidney damage [9]. According to current recommendations of the Kidney Disease: Improving Global Outcomes Work Group, the treatment of patients with T2DM includes lifestyle measures as an important basic therapy, followed by renin-angiotensin system inhibitors, sodium glucose transport protein 2 inhibitors (SGLT-2i), statins, and metformin as first-line drug therapies. Glucagon-like peptide-1 receptor agonists are recommended if SGLT-2i and metformin do not achieve sufficient glycemic control. For patients at high risk of progression to CKD and cardiovascular events despite standard therapies, a nonsteroidal mineralocorticoid receptor antagonist is recommended in addition to baseline therapy.

In order to effectively implement these therapeutic measures to delay progression, early identification of patients with diabetes at increased risk for renal disease is crucial. National and international guidelines recommend to determine the eGFR on the basis of serum creatinine determination and albuminuria using the urine albumin-to-creatinine ratio (UACR) at least once a year in patients with T2DM [8,9,14-18]. In patients diagnosed with CKD, these parameters should be determined up to 4 times a year, depending on the severity and progression risk [9].

However, data on the prevalence of CKD in patients with T2DM in Germany are sparse. Similarly, data on longitudinally eGRF and UACR measurements are limited, as there are only few studies of patients with T2DM and CKD in a real-world clinical setting [19-21]. 

A major aim of the study presented here is to minimize the data gap on the prevalence of CKD in patients with T2DM as well as to assess risk factors, satisfaction with treatment management, and quality of life of patients with T2DM over 12 months. SMART-Finder is the first study exclusively using data provided directly by patients via an adherence-supporting app for this purpose.

### Methods and Objectives

#### Objectives

The primary objective of this study is to investigate the proportion of patients with T2DM with elevated UACR levels (albumin-to-creatinine ratio [ACR] stadium A2 and A3) at baseline and after 12 (±3) months. Secondary objectives include investigating the proportion of patients who remain in or switch to another ACR classification category after 12 months as well as the proportion of patients without UACR-screening data, the description of patient characteristics stratified by hypertension and nephrotoxic comedication status, and information on quality of life for patients with T2DM and relevant subgroups. Further secondary endpoints include the description of disease awareness and medication adherence rates among patients with regard to blood pressure control and antidiabetes drugs. Medication adherence is defined as the number of patients who are compliant and persistent during the observation period, that is, who document at least 80% of their planned medication in the MyTherapy app (compliance) and do not stop taking their planned medication during the observation period (persistence).

#### Study Design

This is a single-arm, retrospective/prospective, observational, digital, patient-centered cohort study in Germany. Both recruitment for this study and documentation of the data are carried out without the assistance of a health care professional (HCP), but as part of the routine use of the MyTherapy app (smartpatient GmbH) by patients with T2DM who document their data in the MyTherapy app using their own smartphone or tablet.

This study is neither intended nor designed to record safety-related data regarding the treatment with T2DM drugs or the use of the MyTherapy app. However, the Diabetes Treatment Satisfaction Questionnaire (DTSQ) questionnaire, which is part of the study electronic case report form (eCRF), captures hypo- and hyperglycemia. Adverse events (AEs) indicative of hypo- or hyperglycemia that may be recorded there, as well as Bayer drug-related AEs recorded in the context of this study, are forwarded from smartpatient, the owner of the MyTherapy app, to the sponsor as a cumulative listing after the end of the study. Furthermore, patients are asked in the patient information of this study to contact HCPs in case of any AEs. For direct reporting, patients are referred to specific web pages [22,23], which is also accessible via an in-app link from the MyTherapy app.

#### Ethics Approval

Ethics approval for this study was granted by the Ethics Committee of the North Rhine Medical Association (Ärztekammer Nordrhein) on October 13, 2022 (approval number 2022263).

Patient consent for the use of personal data is required for participation in the study. The patient information form explains in detail which data will be used for the study and obtains consent for the use of pseudonymized data.

Once the eCRF, including questions on UACR, is completed, patients will receive two €10 (US $10.8) vouchers for Shop Apotheke as compensation for their time spent on data documentation, 1 for completed questionnaires at baseline and 1 after follow-up.

#### Study Population

The study population consists of adult patients with T2DM in Germany using the MyTherapy app to document their medication intake and monitor adherence. Patients with a self-reported diagnosis of type 1 diabetes mellitus and patients who are treating their diabetes with an insulin pump are not eligible.

#### MyTherapy App

**Figure 1:**
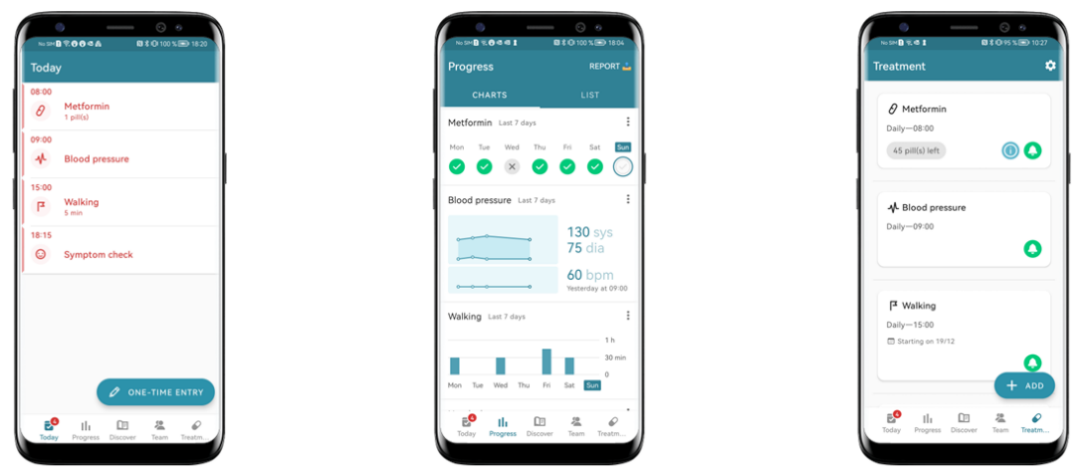
English version of the MyTherapy app sections “Today,” “Progress,” and “Treatment.”.

The MyTherapy app is a widely used medication management app (over 8 Mio Downloads, available in more than 30 languages) that helps patients manage their treatment-related tasks and other health topics, including comorbidities. The application allows the user to track medication intakes and activities and to review them over time. For this purpose, the application translates both simple and complex therapies into a daily list of “To do’s,” which are either related to “Medication,” “Measurement,” “Activity,” or “Symptom check” (Figure 1). The app then supports the user, by reminding them of their health-related tasks; each user is asked to clear their daily list of “To do’s” and is rewarded with a picture of the day afterward. App users can access all tracked events over time in the “Progress” section or request a printable report to be sent to their email address, which can be printed and brought to the next doctor's visit. Studies have already shown that the app strengthens adherence to medication and the doctor-patient relationship in patients with T2DM [24,25].

The app can also, as in the context of this study, be extended by additional functionalities, such as educational content or surveys. In this case, the user benefits from all features of the basic MyTherapy app, while additionally having access to the extension. For this study, specific content (ie, video, patient information on the study) is provided, users are enabled to give informed consent to participate in the study, and a standardized eCRF is shared with and can be completed by MyTherapy app users who participate in the study.

As the MyTherapy app is routinely used by about 13,000 patients with T2DM in Germany to document their medication adherence and to monitor adherence to self-determined goals [24], it provides a cohort of patients suitable for this study. Therefore, usage of the MyTherapy app enables efficient recruitment of eligible patients and collection of relevant retrospective and prospective data with low operational effort.

#### Patient Journey and Study Procedure

##### Recruitment and Study Inclusion

Recruitment for this study is exclusively conducted via the MyTherapy app. All users of the MyTherapy app who have set up reminders for a diabetes medication automatically receive an invitation to participate in this study via push notification during their routine app use as well as a reminder 2 weeks later. An initial question further helps to identify the users with T2DM. In this context, the following medications or medical devices are considered for the initial selection of the target group: metformin, acarbose, dipeptidylpeptidase-4 inhibitor, glucagon-like peptide-1 receptor antagonist, renin-angiotensin system inhibitor, SGLT-2i, basal insulin, nonsteroidal mineralocorticoid receptor antagonist, sulfonylurea, and blood glucose test strips. Interested patients can find a video in German about the etiology of CKD and the importance of regular screening, as well as key information about the SMART-Finder study within the “Discover” section (available as Multimedia Appendix 1). After clicking on the push notification, users access information in German about the background and scope of this study and are able to access, read, and agree to the consent form for participation in this study.

##### Self-reporting of Data

All data will be entered by the patients themselves via the MyTherapy app, both as part of routine use and on a specifically developed eCRF in German. After completing the patient informed consent, an eCRF including 4 questionnaires is provided and can be completed directly within the app: One questionnaire on demographic and general data, 1 on laboratory values, and 2 on quality-of-life aspects. The 2 quality-of-life questionnaires are standardized and validated (EuroQol Five Dimensions Questionnaire [EQ-5D] and DTSQ), both implemented for digital use. Table 1 summarizes variables collected within these 4 questionnaires of the eCRF. Multimedia Appendix 2 provides a detailed description of all variables, including their possible response options and ranges, collected in this study.

**Table 1:** Listing of variables collected in the electronic case report form.

Questionnaire/topic	Variables
General data	Name, zip code, year of birth, age, gender, weight (kg), height (cm), smoking habit; last systolic blood pressure, and last diastolic blood pressure
Laboratory values	UACR^a^, eGFR^b^, HbA_1c_^c^, and serum fasting glucose level
QoL I (DTSQ^d^)	Satisfaction with current treatment; signs of hypoglycemia, signs of hyperglycemia; feasibility of treatment; satisfaction with the flexibility of actual treatment; satisfaction with own knowledge about the disease; recommendation of own treatment to others; and satisfaction with the continuation of current treatment
QoL II (EQ-5D-5L)	Mobility, self-care, usual activities, pain/discomfort, and anxiety/depression

^a^UACR: urine-albumin-creatinine-ratio.

^b^eGFR: estimated glomerular filtration rate.

^c^HbA_1c_: hemoglobin A_1c_.

^d^DTSQ: Diabetes Treatment Satisfaction Questionnaire.

Apart from demographic and general data, all questionnaires should be filled in twice by patients: first at baseline documentation and then 12 (±3) months after the initial UACR measurement (“end-of-observation documentation”). To remind participants of this second data entry, users receive an automatically generated push message.

During the first data entry (baseline documentation), patients are asked to document their most recent available lab values of the last 12 months in the eCRF for UACR, eGFR, HbA_1c_ (hemoglobin 1Ac), and blood pressure. If patients do not know these lab values, they are encouraged to request them from their treating physician. For this purpose, a document is provided via the “Discover” section in the app, which can be shared via email or can be printed to inform the treating physicians about lab values that are required and may still need to be collected.

Overall, laboratory values for baseline documentation can be recorded retrospectively for up to 12 months and prospectively for 3 months after giving informed consent for this study. In addition, patients also have to document fasting blood glucose values, smoking status, and aspects of their quality of life and disease awareness within the other questionnaires of the eCRF as part of this baseline documentation.

The “end of observation documentation” takes place 12 (±3) months after the first documented UACR measurement. Accordingly, the observation period (the period between the 2 UACR screenings) for each patient is 12 (±3) months; however, the period between providing informed consent and the end of observation may be less than 12 months due to the possibility of retrospectively collected UACR baseline data.

Each patient may withdraw from the study at any time and without giving a reason. In this case, no further study data will be collected.

#### Statistical Considerations

##### Sample Size

Based on approximately 13,000 MyTherapy app users with T2DM in Germany and recruitment rates from a comparable study focusing on patient adherence and physician-patient relations, participation of up to 5000 patients is expected [24].

##### Statistical Analysis

All variables are analyzed descriptively with appropriate statistical methods: categorical variables by (absolute and relative) frequency tables, frequencies, and continuous variables by sample statistics (ie, mean, standard deviation, minimum, median, quartiles, and maximum). Continuous variables are described by absolute value and as a change from baseline per analysis time point, if applicable. All analyses are performed for the total study population (overall analysis) and stratified by subgroups (eg, age, gender, and blood pressure), whenever reasonable.

#### Data Management and Quality

Data sources are limited to the MyTherapy app database including data that have been received from the health records of the treating physicians and documented in the eCRF by the patients. MyTherapy users only participate in the study after having signed study-related informed consent. Data are documented by the participating patients themselves. Data are not queried after data entry, but a check for multiple documented patients is performed.

To avoid free-text entries and queries, and to ensure convenient and accurate data entry, the eCRF is designed with all but 2 fields being checkboxes with suggested response options. Furthermore, incomplete questionnaires cannot be saved but must first be completed. While this may result in a higher dropout rate, it contributes considerably to the completeness of the data sets of study completers.

With regard to lab values, data entry is only allowed within certain ranges to minimize errors when transferring data (see Multimedia Appendix 2 for detailed information on acceptable ranges per variable). In addition, as described, patients are encouraged to request missing laboratory values from their treating physician and are supported with a document (Multimedia Appendix 2).

Moreover, with regard to the UACR values, patients are given the opportunity to request missing data over a certain period of time: Patients who state “UACR value not yet measured, but have made a doctor's appointment to have my UACR value measured” are informed twice at intervals of 2 weeks via push message that the UACR values are still missing. Subsequently, they must enter a UACR value or select “UACR value not measured.” Otherwise, the questionnaire is considered as not answered, and the participant drops out of the study.

The category “value not measured” can only be selected for the lab values UACR, eGFR as well as HbA_1c_ and provides important information on how the guideline-compliant assessment of these lab values is implemented in routine clinical practice.

Smartpatient is assigned with the development of eCRFs and their integration into the MyTherapy app, the quality control of data collection, and the transfer of the pseudonymized study database to the clinical research organization Institute Dr Schauerte, Munich, Germany, which performs data analyses and data transfer to Bayer. Digital implementation of the 2 validated and standardized quality of life questionnaires in MyTherapy was reviewed and approved by their respective providers, that is, EuroQoL Research Association, Rotterdam, The Netherlands (for EQ-5D) and Prof Clare Bradley, Health Psychology Research, University of London, Egham, Surrey, UK (for DTSQ).

#### Trial Organization and Regulatory Authorization

This study is sponsored by Bayer Vital GmbH, Leverkusen, Germany. It is performed in collaboration with smartpatient GmbH, Munich, Germany, the provider of the MyTherapy app, a subsidiary of Shop-Apotheke, one of the largest internet-based pharmacies in Germany.

## Results

**Figure 2:**
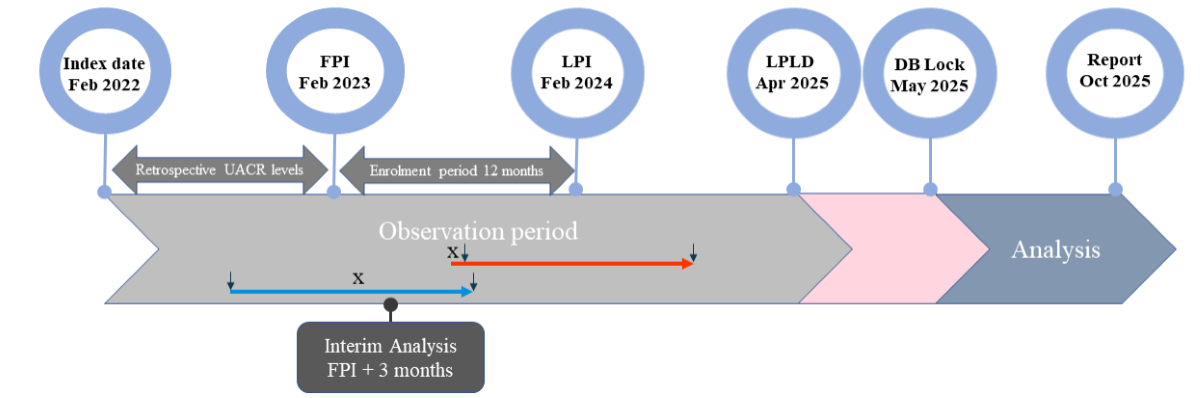
Study time frame illustrating examples for patients with a retrospectively (blue arrow) and a prospectively (orange arrow) measured first UACR level. FPI: first patient in; LPI: last patient in; LPLD: last patient last documentation: DB, database; x = informed consent; ↓: UACR measurement.

The enrollment period of this study is planned from February 2023 (first patient in) until February 2024 (last patient in). During this period, patients accepting the invitation to participate in the study within the MyTherapy app are consecutively enrolled. Enrollment is stopped, after the target sample size of 5000 patients is reached or at the end of the planned enrollment period. Patients dropping out (eg, withdrawal, lost to follow-up) after the end of enrollment are not replaced. The observation period of each individual patient is 12 (±3) months (last patient last documentation: April 2025). An interim analysis is planned 3 months after the inclusion of the first patient and a final analysis after the study end. Figure 2 illustrates the study time frame.

## Discussion

The study design presented here is novel, as it uses data exclusively documented by the patients in an app for the first time to collect information on the prevalence of CKD, risk factors, disease management, and quality of life of patients with T2DM. Since recruitment and data entry for this study are carried out via the MyTherapy app, which is routinely used by approximately 13,000 patients with T2DM in Germany, participation of about 5000 patients is expected. This provides a solid database to answer several scientific questions. This study is the first to estimate the proportion of patients with CKD or at high risk of developing CKD among patients with T2DM using UACR data documented exclusively by patients in the app. Also, any change in disease severity over the course of 12 months is identified in this way. In this context, it is a strength of the cohort study design presented here that the required laboratory values and especially UACR values are collected by HCPs and provided to their patients for entry into the app. It is expected that the study will make an important contribution to minimize the existing data gap in Germany regarding the prevalence of CKD in T2DM patients and contribute to a better understanding of its occurrence, severity, and risk of progression.

In addition, this study will also provide key data on the disease management and health care situation of patients with T2DM in routine clinical practice in Germany. For example, adherence data of (co-)medications reported by patients in the MyTherapy app as well as the identification of treatment despite contraindication (taking nephrotoxic drugs) will provide important insights into therapy implementation in clinical routine.

Another key aspect is that the proportion of patients with T2DM who receive regular, guideline-compliant UACR and eGFR measurements at least once a year will be determined. To date, only limited data are available in this regard, which suggest a certain discrepancy with recommended annual screening and at least hesitant implementation of guideline-based diagnostics with some potential for improvement. A recent study on adherence to treatment guidelines in Germany, based on combined data from the *Diabetes Patienten Verlaufsdokumentation* and *Diabetes Versorgungsevaluation* databases, showed that only 49.1% of 116,747 patients with T2DM received annual CKD screening measurements [19]. Studies from Finland and Australia indicate similar results [20,21].

Considering that CKD screening in patients with T2DM is not yet universally implemented in clinical practice [19-21], another crucial aspect of this app-based study is the fact that it raises awareness of CKD risk in patients with T2DM and also fosters their self-responsibility and health literacy by pointing out the guideline-recommended UACR and eGRF screening of at least once a year. Furthermore, by providing study participants with a printable document for discussion with their treating physician in case of missing lab values, patients are empowered to take care of their disease themselves to a certain extent. For the future, this study also offers the possibility to establish a systematic, app-based early screening for CKD or other fields or indications.

Overall, the study aims to raise awareness of guideline-based management of patients with T2DM, both among physicians and patients and may help to improve early detection of kidney disease in patients with T2DM—a fundamental prerequisite for timely, guideline-based therapy that can prevent or delay disease progression and help to avoid end-stage renal disease.

Limitations of this study include, first, the fact that UACR values cannot be self-assessed by the patients, as the test strips are only approved for use by a health care professional. Even if a printable document is accessible to support medical consultation, it may still pose a barrier for some patients with T2DM to arrange a medical appointment and ask the doctor to collect this value. Therefore, missing UACR values might be a concern. On the other hand, the number of missing UACR values provides important insights into disease management and guideline adherence in Germany. Second, with a follow-up of 12 months, “digital” attrition, that is, a decreasing adherence to the app, is also to be expected, as its regular use may be challenging and may cause dropouts. Third, potential errors in data transmission are also a limitation as patients document values themselves without the possibility of querying them. However, efforts are undertaken to minimize transmission errors by permitting data entry only within a certain range (see Multimedia Appendix 2 for detailed information on the permitted ranges per variable). Fourth, the study population consists of patients with T2DM routinely using the MyTherapy app to review self-defined health goals and to support their medication adherence—and thus represents a biased subpopulation of health-conscious and technology-savvy patients with T2DM. Accordingly, it may be expected that the UACR screening rate determined in this study may rather represent an overestimation. Nevertheless, it can be assumed that, due to the recruitment and consecutive enrollment among health app users with only very limited exclusion criteria, the study population covered in this study is considerably more “heterogeneous” in terms of health than that in controlled, randomized clinical trials and can therefore provide a more realistic picture of the disease and management situation of T2DM patients in Germany.

In conclusion, it can be assumed that the study will provide important insights into the current disease management of patients with T2DM in everyday clinical practice in Germany as well as into the prevalence of patients with elevated UACR values in this T2DM cohort and will support guideline-based care of the participating patients.

## Appendix

Multimedia Appendix 1 Introduction Video presented in MyTherapy app.

Multimedia Appendix 2 Variables, including question type, possible response options, and ranges collected in SMART-Finder.

## Abbreviations

ACR: albumin-to-creatinine-ratio
AE: adverse event
CKD: chronic kidney disease
DTSQ: Diabetes Treatment Satisfaction Questionnaire
eCRF: electronic case report form
eGFR: estimated glomerular filtration rate
EQ-5D: EuroQol Five Dimensions Questionnaire
HCP: health care professional
HbA_1c_: hemoglobin A_1c_
SGLT-2i: sodium-glucose transport protein 2 inhibitors
T2DM: type 2 diabetes mellitus
UACR: urine albumin-to-creatinine ratio
